# Weight management in cardiology practice: challenges and opportunities

**DOI:** 10.1007/s12471-026-02075-1

**Published:** 2026-09-22

**Authors:** Maarten J. G. Leening, Fabrice M. A. C. Martens, Martin E. W. Hemels

**Affiliations:** 1 Departments of Cardiology, Radiology, and Epidemiology, Erasmus MC—University Medical Center Rotterdam, Rotterdam, The Netherlands; 2 Department of Cardiology, Amsterdam University Medical Center, Amsterdam, The Netherlands; 3 Department of Cardiology, Rijnstate Hospital, Arnhem, The Netherlands; 4 Department of Cardiology, Radboud University Medical Center, Nijmegen, The Netherlands

**Keywords:** Obesity, Cardiovascular Disease, Weight Management, Glucagon-like Peptide‑1 (GLP-1) Agonists, Prevention

## Abstract

Obesity poses a clear challenge to the cardiovascular landscape. In this viewpoint, we advocate that we need to prepare for the practical implementation of weight management in Dutch cardiology practice by 1) educating ourselves and our peers on obesity and its treatment thereof, 2) considering how to facilitate the appropriate amount of clinical follow-up, and 3) establish local arrangements with all health care professionals involved in the chain of lifelong atherosclerotic cardiovascular disease risk management. Pending the results of ongoing clinical trials involving weight management therapies tested for other cardiovascular conditions (e.g. heart failure and atrial fibrillation), and the evolving landscape of health care budgets and capacity, it is to be expected that the role of weight management therapies will become increasingly prominent in cardiology practice: we therefore argue it would be wise to make an early start with systematic implementation.

Obesity poses a clear challenge to the cardiovascular landscape. An abundance of genetic and clinical studies have pointed to a causal role for obesity in the development of atherosclerosis, heart failure, and atrial fibrillation: either directly or indirectly through traditional risk factors like diabetes, lipids, hypertension, and sleep apnea [1–3]. Moreover, once an individual with obesity develops cardiovascular disease, obesity can limit on the treatment options, particularly invasive procedures, and come with a higher risk of numerous treatment-related complications. Risk factors are often more challenging to control, disease progression is faster, and quality of life is more severely impaired in patients with obesity [1–3]. As such, obesity is omnipresent in everyday clinical cardiology practice. This has become even more apparent with the recent advent of weight management with medication and lifestyle modification. In this viewpoint, we advocate the implementation of weight management in Dutch cardiology practice.

  Smoking rates have been steadily declining over the past decades, accompanied by greater awareness and an expanding armamentarium of treatments for hypertension, lipids, and diabetes [4, 5]. This has contributed to a spectacular decline in the incidence and mortality of cardiovascular disease over the past 70 years [4]. The prevalence of obesity, however, has been steadily increasing over time, with currently more than 75% of patients with chronic coronary artery disease in the Netherlands being overweight and more than one in four being obese [6].

Prevention of obesity, starting in early childhood, is key to long-term strategies to reduce the burden of obesity in the general population. However, as it stands, many adults already face the consequences of obesity and need dedicated care to manage their weight. Until recently, lifestyle management and bariatric surgery have been the mainstays of obesity management at the individual patient level. Lifestyle programs have been effective for some patients, but have limited long-term effects for many [7, 8]. Bariatric and metabolic surgery generally results in substantial weight reduction, risk factor control, and is ultimately associated with cardiovascular risk reduction in most operated patients [9], but is only utilized sparsely from a population perspective. As such, besides lifestyle programs and surgery, there is a need for highly effective, evidence-based weight management interventions applicable to the wider population.

## Opportunities

Incretin-based therapies, spearheaded by glucagon-like peptide‑1 (GLP-1) receptor agonists and more recently followed by combination therapies with other incretins and amylin analogs, have changed the landscape of obesity management [10]. With over 2 decades of clinical experience in patients with diabetes, more recently the field has transitioned from diabetes to obesity. Multiple cardiovascular outcome trials have consistently demonstrated a reduction in major adverse cardiovascular events (MACE) with GLP‑1 agonists compared to placebo among individuals with diabetes, which was on top of usual care including recommendations on diet and exercise [11, 12]. In 2023, the SELECT trial demonstrated lower risks of MACE with semaglutide compared to placebo among individuals without diabetes with established atherosclerotic cardiovascular disease (ASCVD) and a body mass index ≥ 27 kg/m^2^ [13]. These findings address an important unmet need and provide an opportunity to address obesity-related cardiovascular risk among many of our patients.

It is expected that GLP‑1 agonists will be made available for weight-related risk management in patients with ASCVD, and as such be added to the armamentarium of the cardiologist in adjunct to lifestyle management strategies. Aside from ASCVD prevention, incretin-based therapies have been shown effective in improving symptoms or outcomes in overweight or obese patients with type 2 diabetes [11], chronic kidney disease [14], metabolic dysfunction-associated steatotic liver disease [15, 16], obstructive sleep apnea [17], knee osteoarthritis [18], and heart failure with preserved ejection fraction [19, 20]. As such, a vast number of patients stand to benefit from these therapies [21, 22], and hence important decisions need to be made by regulatory bodies regarding healthcare budgets, cost-effective implementation, and the governance of these therapies. As a medical community, we need to facilitate these discussions surrounding these decisions with guidance on where the largest unmet needs exists among our patients.

## Challenges

Aside from the aforementioned clinical benefits, weight management present inherent challenges. In the SELECT trial, high-dose semaglutide was generally well tolerated, yet one in ten participants discontinued semaglutide because of gastrointestinal intolerance, and in total one in six participants discontinued medication during a median follow-up of 3 years [13]. In a real-world setting, almost two thirds of patients discontinue GLP‑1 receptor agonists within the first year of initiation [23], likely reflecting not only tolerability, but also non-medical determinants such as out-of-pocket costs. Similar challenges exist for combined lifestyle intervention (GLI) programs: based on a recently published report from the Dutch National Institute for Public Health and the Environment (RIVM), nine out of ten patients with an intake visit proceed to enter the program, but only six out of ten complete the full 2‑year program [8].

Moreover, weight loss with incretin-based therapies in addition to lifestyle advice generally reaches a plateau after approximately one year of treatment. Withdrawal of incretin-based therapies, after the weight plateau has been reached, yields sustained weight loss and associated cardiometabolic benefits for only a minority of patients [23–25]. As such, most patients will require some form of long-term maintenance therapy to avoid so-called “weight cycling” and the “yo-yo effect” [23]. Weaning schemes and optimal dosing for those who require maintenance therapy are areas of ongoing research.

## A call to action

We need to prepare for the practical implementation of this new axis of care in clinical cardiology by 1) educating ourselves and our peers on obesity and its treatment thereof, 2) considering how to facilitate the appropriate amount of clinical follow-up, and 3) establish local arrangements with all health care professionals involved in the chain of lifelong ASCVD risk management.

The introduction of weight management therapies in cardiology practice will likely be a collaborative endeavor. We, as a cardiology community, have great opportunities to learn from our colleagues with over a decade of experience prescribing GLP‑1 agonists to patients with diabetes. Although at this point it is unclear what the updated reimbursement criteria for weight management therapies will be in the Netherlands, it is obvious that incretin-based therapies will become more widely available in everyday clinical cardiology practice. Some estimates suggest that half of all patients with coronary artery disease will be eligible [21, 22]. This poses a significant challenge to the logistics of our everyday clinical practice and requires forward thinking: access and referral to lifestyle intervention programs will require local arrangements, whereas dose escalation of medication and guidance of patients with adverse effects will require follow-up. We as cardiologists cannot rely on other health care professionals to routinely manage obesity-related cardiovascular risks. After all, weight management should be part of a holistic cardiovascular risk management approach.

Pending the results of ongoing outcome trials involving incretins tested for other cardiovascular conditions (e.g. heart failure and atrial fibrillation), and the evolving landscape of health care budgets and capacity, it is to be expected that the role of weight management therapies will become increasingly prominent in cardiology practice: we would therefore be wise to make an early start with systematic implementation.

## Data Availability

All data supporting the findings of this work are included in the article.

## References

[1] Powell-Wiley TM, Poirier P, Burke LE, et al. Obesity and cardiovascular disease: a scientific statement from the American Heart Association. Circulation. 2021;143(21):e984–e1010. 10.1161/CIR.0000000000000973 PMC8493650 33882682

[2] Lopez-Jimenez F, Almahmeed W, Bays H, et al. Obesity and cardiovascular disease: mechanistic insights and management strategies. A joint position paper by the World Heart Federation and World Obesity Federation. Eur J Prev Cardiol. 2021;29(17):2218–37. 10.1093/eurjpc/zwac187 36007112

[3] Koskinas KC, Van Craenenbroecket EM, Antoniades C, et al. Obesity and cardiovascular disease: an ESC clinical consensus statement. Eur Heart J. 2024;45(38):4063–98. 10.1093/eurheartj/ehae508 39210706

[4] Nabel EG, Braunwald E. A tale of coronary artery disease and myocardial infarction. N Engl J Med. 2012;366(1):54–63. 10.1056/NEJMra1112570 22216842

[5] Laing BY, Katz MH, Tarone RE, et al. Coronary arteries, myocardial infarction, and history. N Engl J Med. 2012;366(13):1258–60. 10.1056/NEJMc1201171 22455432

[6] Wood DA, Jennings CS, Kotseva K, et al. ESC Guidelines on prevention and rehabilitation in coronary heart disease and current practice: the EUROASPIRE VI survey. Eur Heart J. 2026; ehag741. 10.1093/eurheartj/ehag741. 42666091

[7] de Brauw LM. De gecombineerde leefstijlinterventie: een papieren tijger in de strijd tegen obesitas? Ned Tijdschr Geneeskd. 2021;165:D5885. 33793134

[8] Oosterhoff M, Rotteveel A, Hulshof TA, Rijksinstituut voor Volksgezondheid en Milieu (RIVM), et al. Deelname en uitval bij de gecombineerde leefstijlinterventie (GLI): wie en waarom? rapport 2025-0085. 2025; 10.21945/RIVM-2025-0085.

[9] van Veldhuisen SL, Gorter TM, van Woerden G, et al. Bariatric surgery and cardiovascular disease: a systematic review and meta-analysis. Eur Heart J. 2022;43(20):1955–69. 10.1093/eurheartj/ehac071 PMC9123239 35243488

[10] Nauck MA, Tuttle KR, Tschöp MH, Blüher M. Glucagon-like receptor agonists and next-generation incretin-based medications: metabolic, cardiovascular, and renal benefits. Lancet. 2026;407(10531):892–908. 10.1016/S0140-6736(25)02105-1 41547366

[11] Lee MMY, Sattar N, Pop-Busui R, et al. Cardiovascular and kidney outcomes and mortality with long-acting injectable and oral glucagon-like peptide 1 receptor agonists in individuals with type 2 diabetes: a systematic review and meta-analysis of randomized trials. Diabetes Care. 2025;48(5):846–59. 10.2337/dc25-0241 40156846

[12] Martens FMAC, Visseren FLJ, Westerink J, et al. 2023 European Society of Cardiology guidelines for the management of cardiovascular disease in patients with diabetes: statement of endorsement by the NVVC. Neth Heart J. 2025;33(7-8):216–25. 10.1007/s12471-025-01967-y PMC12274173 40634662

[13] Lincoff AM, Brown-Frandsen K, Colhoun KM, et al. Semaglutide and cardiovascular outcomes in obesity without diabetes. N Engl J Med. 2023;389(24):2221–32. 10.1056/NEJMoa2307563 37952131

[14] Chen JY, Hsu TW, Liu JH, et al. Kidney and cardiovascular outcomes among patients with CKD receiving GLP‑1 receptor agonists: a systematic review and meta-analysis of randomized trials. Am J Kidney Dis. 2025;85(5):555–69. 10.1053/j.ajkd.2024.11.013 39863261

[15] Loomba R, Hartman ML, Lawitz EJ, et al. Tirzepatide for metabolic dysfunction-associated steatohepatitis with liver fibrosis. N Engl J Med. 2024;391(4):299–310. 10.1056/NEJMoa2401943 38856224

[16] Sanyal AJ, Newsome PN, Kliers I, et al. Phase 3 trial of semaglutide in metabolic dysfunction-associated steatohepatitis. N Engl J Med. 2025;392(21):2089–99. 10.1056/NEJMoa2413258 40305708

[17] Malhotra A, Grunstein RR, Fietze I, et al. Tirzepatide for the treatment of obstructive sleep apnea and obesity. N Engl J Med. 2024;391(13):1193–205. 10.1056/NEJMoa2404881 PMC11598664 38912654

[18] Bliddal H, Bays H, Czernichow S, et al. Once-weekly semaglutide in persons with obesity and knee osteoarthritis. N Engl J Med. 2024;391(17):1573–83. 10.1056/NEJMoa2403664 39476339

[19] Kosiborod MN, Abildstrøm SZ, Borlaug BA, et al. Semaglutide in patients with obesity-related heart failure and preserved ejection fraction. N Engl J Med. 2023;389(12):1069–84. 10.1056/NEJMoa2306963 37622681

[20] Packer M, Zile MR, Kramer CM, et al. Tirzepatide for heart failure with preserved ejection fraction and obesity. N Engl J Med. 2025;392(5):427–37. 10.1056/NEJMoa2410027 39555826

[21] Hansen MK, Olesen KKW, Gyldenkerne C, et al. Eligibility for and preventive potential of semaglutide in overweight and obese patients with myocardial infarction. J Am Coll Cardiol. 2024;83(9):956–8. 10.1016/j.jacc.2023.12.029 38418010

[22] Shi I, Khan SS, Yeh RW, et al. Semaglutide eligibility across all current indications for US adults. JAMA Cardiol. 2025;10(1):96–8. 10.1001/jamacardio.2024.4657 PMC11574724 39556764

[23] Moiz A, Filion KB, Knäuper B, et al. Weight maintenance after discontinuation of GLP‑1 therapies. eClinicalMedicine. 2026;96:103992. 10.1016/j.eclinm.2026.103992 PMC13240724 42256676

[24] Wilding JPH, Batterham RL, Davies M, et al. Weight regain and cardiometabolic effects after withdrawal of semaglutide: the STEP 1 trial extension. Diabetes Obes Metab. 2022;24(8):1553–64. 10.1111/dom.14725 PMC9542252 35441470

[25] Horn DB, Aronne LJ, Wharton S, et al. Tirzepatide for maintenance of bodyweight reduction in people with obesity in the USA (SURMOUNT-MAINTAIN): a multicentre, double-blind, randomised, placebo-controlled trial. Lancet. 2026;407(10545):2305–18. 10.1016/S0140-6736(26)00656-2 42119587

